# Evaluation of the influence of genetic variants in Cereblon gene on the response to the treatment of erythema nodosum leprosum with thalidomide

**DOI:** 10.1590/0074-02760220039

**Published:** 2022-11-14

**Authors:** Perpétua do Socorro Silva Costa, Miriãn Ferrão Maciel-Fiuza, Thayne Woycinck Kowalski, Lucas Rosa Fraga, Mariléa Furtado Feira, Luís Marcelo Aranha Camargo, Daniele Iop de Oliveira Caldoncelli, Maria Irismar da Silva Silveira, Lavínia Schuler-Faccini, Fernanda Sales Luiz Vianna

**Affiliations:** 1 Universidade Federal do Rio Grande do Sul, Programa de Pós-Graduação em Genética e Biologia Molecular, Porto Alegre, RS, Brasil; 2 Instituto Nacional de Genética Médica Populacional, Porto Alegre, RS, Brasil; 3 Universidade Federal do Maranhão, Centro de Ciências de Imperatriz, Coordenação de Enfermagem, Imperatriz, MA, Brasil; 4 Hospital de Clínicas de Porto Alegre, Centro de Pesquisa Experimental, Laboratório de Medicina Genômica, Porto Alegre, RS, Brasil; 5 Universidade Federal do Rio Grande do Sul, Departamento de Genética, Laboratório de Imunologia e Imunogenética, Porto Alegre, RS, Brasil; 6 Hospital de Clínicas de Porto Alegre, Serviço de Genética Médica, Sistema de Informações sobre Agentes Teratogênicos, Porto Alegre, RS, Brasil; 7 Universidade Federal do Rio Grande do Sul, Programa de Pós-Graduação em Ciências Médicas, Porto Alegre, Brasil; 8 Universidade Federal do Rio Grande do Sul, Instituto de Ciências Básicas da Saúde, Departamento de Ciências Morfológicas, Porto Alegre, RS, Brasil; 9 Universidade de São Paulo, Instituto de Ciências Biomédicas-5, Monte Negro, RO, Brasil; 10 Centro de Pesquisa em Medicina Tropical, Porto Velho, RO, Brasil; 11 Instituto Nacional de Ciência e Tecnologia de Epidemiologia da Amazônia Ocidental, Porto Velho, RO, Brasil; 12 Centro Universitário São Lucas, Departamento de Medicina, Porto Velho, RO, Brasil; 13 Centro de Referência Nacional em Dermatologia Sanitária Dona Libânia, Fortaleza, CE, Brasil

**Keywords:** CRBN, leprosy, pharmacogenomics, pharmacogenetics, personalised medicine

## Abstract

**BACKGROUND:**

Erythema nodosum leprosum (ENL) is an acute and systemic inflammatory reaction of leprosy characterised by painful nodules and involvement of various organs. Thalidomide is an immunomodulatory and anti-inflammatory drug currently used to treat this condition. Cereblon (CRBN) protein is the primary target of thalidomide, and it has been pointed out as necessary for the efficacy of this drug in others therapeutics settings.

**OBJECTIVES:**

In this study, we aimed to evaluate the influence of *CRBN* gene variants on the dose of thalidomide as well as its adverse effects during treatment of ENL.

**METHODS:**

A total of 103 ENL patients in treatment with thalidomide were included in this study. DNA samples were obtained from saliva and molecular analysis of *CRBN* gene were performed to investigate the variants rs1620675, rs1672770 and rs4183. Different genotypes of *CRBN* variants were evaluated in relation to their influence on the dose of thalidomide and on the occurrence of adverse effects.

**FINDINGS:**

No association was found between *CRBN* variants and thalidomide dose variation. However, the genotypes of rs1672770 showed association with gastrointestinal effects (p = 0.040). Moreover, the haplotype DEL/C/T (rs4183/rs1672770/rs1620675) was also associated with gastrointestinal adverse effects (p = 0.015).

**MAIN CONCLUSIONS:**

Our results show that *CRBN* variants affect the treatment of ENH with thalidomide, especially on the adverse effects related to the drug.

ENL is a difficult to manage leprosy complication and a potentially disabling condition.^1^ ^,^ ^2^ It is an inflammatory reaction characterised by painful nodules on the skin that can ulcerate and by systemic involvement with fever and general malaise and effects on various organs.^2^ ^,^ ^3^ The reaction affects patients with borderline lepromatous (BL) leprosy and lepromatous leprosy (LL) that are associated with a higher bacillary load.^1^ ^,^ ^2^

Thalidomide is effective in treating ENL, rapidly reducing symptoms, such as fever and night sweats, and improving skin lesions.^4^ ^,^ ^5^ Thalidomide is a glutamic acid derivative produced initially in the 1950s as an anticonvulsant and subsequently used as a sedative and antiemetic in early pregnancy.^4^ ^,^ ^6^ However, its teratogenic effect became known when reports of birth defects in children born to mothers who used thalidomide during pregnancy appeared in 1961.^7^ ^,^ ^8^ Thalidomide was soon withdrawn from the market; however its efficacy in the treatment of erythema nodosum leprosum (ENL) was accidently discovered few years later, bringing it back for the clinical use even within strict regulations.^5^ By 1999, thalidomide was also proved to be effective in the treatment of multiple myeloma (MM)^9^ ^,^ ^10^ and later for other conditions.

The effectiveness of thalidomide in ENL is initially due to its action on tumour necrosis factor (TNF)-α, but other mechanisms may contribute to its anti-inflammatory effect.^11^ ^,^ ^12^ Cereblon protein (CRBN) was described as the primary target of thalidomide, being involved also with the teratogenic potential of the drug.^13^ CRBN is part of an E3-ubiquitin ligase complex (CRL4^CRBN^), acting as a substrate receptor that recognises specific targets for ubiquitination, leading to further degradation by the ubiquitin-proteasome system.^14^ More recently, it was shown that CRBN is necessary for the efficacy of thalidomide and its analogs lenalidomide and pomalidomide [named immunomodulatory drugs (IMiDs)] in the treatment of MM.^15^ ^-^ ^19^ Several studies have investigated the thalidomide-CRBN interaction with regard to the teratogenic, immunomodulatory and therapeutic effects of the drug. However, there are no studies on the effect of the thalidomide-CRBN interaction on ENL.

*CRBN* gene encodes a protein 442 amino acids long, has 11 exons (NM_016302) and is highly conserved; hence, polymorphisms in its coding regions are rare. Therefore, some studies have analysed non-coding regions of the gene that may be associated with the control of gene expression.^20^ ^-^ ^22^ Three variants that flank the exons encoding the thalidomide-binding region of CRBN have been identified by bioinformatics tools as possible modulators of splicing sites with the potential to affect either the expression or activity of the protein.^23^ They are located in regions adjacent to the region encoding the portion of the protein that binds thalidomide, an A>C substitution (rs1620675) and a G>A substitution (rs1672770), both in intron 10, and an insertion/deletion (INS/DEL) of four nucleotides (-/GTTA) in the 3’UTR region adjacent to exon 11 (rs4183). Considering the role of CRBN on thalidomide actions and that these variants might affect the function, activity or expression of the protein, we evaluated the influence of these three variants in *CRBN* on the variation of the dose of thalidomide and the occurrence of adverse effects on the treatment of ENL. 

## SUBJECTS AND METHODS

*Sample -* The sample consisted of 103 ENL patients who were selected from National Reference Center of Sanitary Dermatology Dona Libânia in Fortaleza (state of Ceará, Brazil), Humanized Reference Center of Sanitary Dermatology in Imperatriz and Aquiles Lisboa Hospital in São Luís (Maranhão State) in northeast Brazil, and from the Dermatology Ambulatory of the University of São Paulo in Monte Negro (state of Rondônia, Brazil) in north Brazil. 

The patients used thalidomide at different doses and had a follow-up of up to six visits (average of 3.6 months). Data from up to six consultations annotated in the patient’s medical record were analysed with the collection of clinical and demographic information, including sex, age and region of origin, history of leprosy (moment of diagnosis and treatment used) and history of ENL (diagnosis, treatment, adverse effects, history of relapse and dose of medications used).

*Genetic analyses -* DNA was extracted from saliva samples using the Oragene DNA Extraction Kit (DNA Genotek, Ottawa, Canada), according to the manufacturer’s instructions. A pair of primer was designed to amplify a fragment of 682 base pairs containing the region encompassing the three studied *CRBN* variants: forward 5’-TGTGGTCTTGGCAACCAGCAATTT-3’ and reverse 5’-ACTGCCGTTCATGCTTGTTTCCT-3’. This region was amplified by polymerase chain reaction (PCR). The fragment obtained was visualised on a 2% agarose gel, purified and sequenced using the same primers.

Sequences were visualised and analysed using CodonCodeAligner^®^, version 3.0.1 (CodonCode Corporation, Dedham, USA). The hg19 sequence deposited in GenBank was used as the reference sequence. When there was doubt about the variant, sequencing was repeated for confirmation.

*Statistical analyses -* Chi-square test was used to evaluate Hardy-Weinberg equilibrium for all polymorphisms. Generalised estimating equations method (GEE) was used to evaluate the influence of *CRBN* variants on thalidomide dose. This method is a repeated measures analysis focused on average changes in response over time and on the impact of covariates on these changes. GEE can model the average response of variables as a linear function of covariates of interest through a transformation or link function and can be used in studies where the data is asymmetric or the data distribution is difficult to verify due to the small-size sample.^24^ ^,^ ^25^ The covariates inserted in the model were place of origin of the patient, concomitant use of multidrug therapy (MDT) for leprosy and use of other medications and other treatments for ENL.

The evaluation of the association of CRBN variants and haplotypes in the occurrence of adverse effects due to thalidomide treatment was based on clinical data using logistic regression. Models with and without correction by gender were used. The INS/C/T (rs4183/rs1672770/rs1620675) haplotype was removed from the association analyses because it presented few events and disturbed the analyses. Peripheral polyneuropathy, although is an adverse effect common to the use of thalidomide, has not been evaluated because it is difficult to distinguish it from polyneuropathy caused by ENL and leprosy. All statistical analyses were performed with SPSS version 20 (www.spss.com).

MLocus tool was used to calculate linkage disequilibrium (LD) for the variants,^26^ and haplotypes were inferred using the Bayesian algorithm of the phase 2.1.1 program.^27^ ^,^ ^28^

*Data availability -* The data analysed during the current study are not publicly available to maintain patient confidentiality. Moreover, this type of request has not been previously approved by participants nor the human research committee. This data could, however, be available (anonymously) from the corresponding author on reasonable request.

*Ethics -* All participants were informed about the research objectives and signed an informed consent form. This study was approved by the Ethics Committee of the Hospital de Clínicas of Porto Alegre under number CAAE 21184413.0.0000.5327. All research was performed in accordance with Brazilian regulations and informed consent was obtained from all participants. This research has been performed in accordance with the Declaration of Helsinki.

## RESULTS

In total, 103 ENL patients were included, being 82 (79.6%) male and 79 (76.7%) presenting LL (Table I). Forty-six (44.7%) patients were using MDT and two of these used an alternative MDT regimen. The mean time of treatment with thalidomide was 226 days.

**TABLE I:** Clinical and demographic characteristics of ENL patients (n = 103)

Characteristic	n (%)
Male	82 (79.6)
MDT for leprosy	46 (44.7)
MDT time in months median (P25/P75)*	4.3 (1.5/10.9)
Other medications	81 (78.6)
Thalidomide dose median (P25/P75)	100 (100/200)
Days of consultation mean (min/max)	108.19 (0/721)
Patient origin	
Northeast Region	89 (86.4)
North Region	14 (13.6)
Leprosy classification	
Borderline-lepromatous	24 (23.3)
Lepromatous-leprosy	79 (76.7)
Adverse effects	
Neurological^*a*^	31 (30.1)
Grastrointestinal^*b*^	18 (17.5)
Musculoskeletal^*c*^	29 (28.2)
Ocular^*d*^	19 (18.4)
Oedema	16 (15.6)
Dermatological^*e*^	2 (1.9)
Fever	11 (10.7)

*a*: paresthesias, dizziness, tremor, neuritis and headache; *b*: diarrhoea, vomiting, gastric fullness and constipation; *c*: myalgia, arthralgia and weakness; *d*: decreased visual acuity and eye irritation; *e*: pruritus, dry skin and hair loss; *: time of multidrug therapy (MDT) when patients developed erythema nodosum leprosum (ENL) (n = 40).

The genotypic distributions were in Hardy-Weinberg equilibrium and the allelic and genotypic frequencies of the polymorphisms are shown in Table II. A LD [rs4183/rs1672770 (r2 = 0.513), rs4183/rs1620675 (r2 = 0.943), rs1672770/rs1620675 (r2 = 0.567)] between the variants studied was identified (see Supplementary Table) and four haplotypes were identified in the sample (Table III). For all single nucleotide polymorphisms (SNPs), there was no association of the thalidomide dose with time of treatment (Table IV). In addition, no association was found between haplotypes and thalidomide dose.

**TABLE II:** Genotypic and allelic frequencies of CRBN polymorphisms in ENL patients on treatment with thalidomide

Polymorphism	Alleles/genotypes	Frequency n (%)
rs1620675	AA	31 (30.1)
	AC	53 (5.5)
	CC	19 (18.4)
	A	115 (55.8)
	C	91 (44.2)
	
rs1672770*	AA	31 (30.1)
	AG	52 (50.5)
	GG	17 (16.5)
	A	114 (55.3)
	G	86 (44.7)
rs4183	INS/INS	19 (18.4)
	INS/DEL	56 (54.4)
	DEL/DEL	28 (27.2)
	INS	94 (45.6)
	DEL	115 (54.4)

DEL: deletion; ENL: erythema nodosum leprosum; INS: insertion; MDT: multidrug therapy; *: n = 100.

**TABLE III:** Haplotype frequencies of *CRBN*

Haplotype	n	(%)
INS/C/T	3	1.5
INS/T/G	92	44.7
DEL/C/T	83	40.3
DEL/T/T	28	13.6

Haplotypes in the following order: rs4183/rs1672770/rs1620675. DEL: deletion; *CRBN*: *cereblon* gene; INS: insertion.

**TABLE IV:** Analysis of interaction between CRBN genotype and time related to estimated thalidomide dose using the GEE model in ENL treatment

Polymorphism	Interaction	p-value
rs1620675	rs1620675	0.079
	Concomitant MDT for leprosy	0.271
	Other medications	0.205
	Region	0.138
	Time	0.001
rs1672770	rs1620675*Time	0.392
	rs1672770	0.229
	Concomitant MDT for leprosy	0.296
	Other medications	0.071
	Region	0.085
	Time	0.001
	rs1672770*Time	0.273
rs4183	rs4183	0.098
	Concomitant MDT for leprosy	0.293
	Other medications	0.208
	Region	0.117
	Time	0.001
	rs4183*Time	0.669

Dependent variable: thalidomide dose. GEE model: region, MDT, other medications, thalidomide dose, genotype, time, genotype*Time). CRBN: cereblon; ENL: erythema nodosum leprosum; GEE: generalised estimating equation model; MDT: multidrug therapy.

In regard to the relationship of *CRBN* variants and adverse effects of the use of thalidomide, it was found associations between the genotypes of rs1672770 (p = 0.040) and gastrointestinal effects, which include diarrhoea, vomiting, nausea, constipation and inappetence (Tables V and VI). Haplotype analysis was performed with all SNPs because only one combination of SNPs showed r2>0.8 (rs4183/rs1620675 with r2 = 0.943). The three polymorphisms were maintained in the analysis because when combining rs4183/rs1672770 or rs1672770/rs1620675, it showed r2<0.8. The analysis of the association between haplotypes and adverse effects also showed an association of gastrointestinal adverse effects (p = 0.015) with haplotype DEL/C/T (rs4183/rs1672770/rs1620675) (Tables VII and VIII). These effects were present in eight patients (9.6%) who carried this haplotype. 

**TABLE V:** Analysis of the association of *CRBN* variants on the occurrence of gastrointestinal adverse effects^*a*^

Polymorphism	OR^*b*^	p-value*	OR^*b*^	p-value**
rs1672770				
AA	REF	-	REF	-
AG	0.325 (0.111-0.951)	0.040	0.040 (0.111-0.948)	0.040
GG	0.131 (0.015-1.115)	0.063	0.127(0.015-1.100)	0.061

Dependent variable: gastrointestinal. *CRBN*: *cereblon* gene; OR: odds ratio; *a*: gastrointestinal adverse effects (diarrhoea, vomiting, nausea, constipation and inappetence); *b*: logistic regression (OR and 95% confidence interval); *: model rs1672770; **: model sex, rs1672770.

**TABLE VI:** Frequency of gastrointestinal adverse effects^*a*^ according to *CRBN* genotypes

Polymorphism	Genotype	Absence (n) (% within effect)	Presence (n) (% within effect)
rs1672770	AA	23 (67.6)	11 (32.4)
	AG	45 (86.5)	7 (13.5)
	GG	16 (94.1)	1 (5.9)

*CRBN*: *cereblon* gene; *a*: gastrointestinal adverse effects (diarrhoea, vomiting, nausea, constipation and inappetence).

**TABLE VII:** Analysis of the association of *CRBN* haplotypes on the occurrence of gastrointestinal adverse effects^*a*^

Haplotype	OR^*b*^	p-value*	OR^*b*^	p-value**
INS/T/G	REF	-	REF	-
DEL/C/T	0.339 (0.142-0.812)	0.015	0.333 (0.138-0.805)	0.015
DEL/T/T	1.061 (0.398-2.827)	0.906	1.022 (0.371-2.818)	0.966

Dependent variable: gastrointestinal. DEL: deletion; *CRBN*: *cereblon* gene; INS: insertion; OR: odds ratio; *a*: gastrointestinal adverse effects (diarrhoea, vomiting, nausea, constipation and inappetence); *b*: logistic regression (OR and 95% confidence interval); *: model haplotypes; **: model sex, haplotypes.

**TABLE VIII:** Frequency of gastrointestinal adverse effects^*a*^ according to *CRBN* haplotypes

Haplotypes	Absence (n) (% within effect)	Presence (n) (% within effect)
INS/T/G	70 (76.1)	22 (23.9)
DEL/C/T	75 (90.4)	8 (9.6)
DEL/T/T	21 (75)	7 (25)

DEL: deletion; *CRBN*: *cereblon* gene; INS: insertion; *a*: gastrointestinal adverse effects (diarrhoea, vomiting, nausea, constipation and inappetence).

## DISCUSSION

This study aimed to identify genetic variants in *CRBN* gene that might influence in response to the treatment of ENL with thalidomide. We identified that the SNP rs1672770 and the haplotype DEL/C/T (rs4183/rs1672770/rs1620675) were associated with the manifestation of adverse gastrointestinal effects.

CRBN acts as a substrate receptor as part of the E3 ubiquitin ligase complex (CRL4^CRBN^), which controls the expression of target proteins by their ubiquitination and degradation. It is necessary for the teratogenic effect of thalidomide and also important for the antiproliferative effect of thalidomide and other IMiDs in MM.^29^ It is postulated that when thalidomide binds to CRBN, it modifies its function causing teratogenic effects by preventing the degradation proteins and/or by creating neosubtrates for ubiquitination and proteasomal degradation, which play a crucial role in embryonic development.^30^ ^,^ ^31^ In the case of MM, thalidomide binding to CRBN promotes recruitment of the neosubstrates Ikaros (IKZF1) and Aiolos (IKZF3) to the ubiquitin-ligase complex, resulting in increased ubiquitination and degradation of these transcription factors in T cells and MM cells.^31^ ^-^ ^33^ In addition, some studies have associated low *CRBN* mRNA expression with poorer clinical response to IMiDs, suggesting a potential role of CRBN as a predictive biomarker for treatment response.^17^ ^,^ ^31^ ^,^ ^32^ ^,^ ^34^ Since the 1990s, it is known that one of the main effects of thalidomide is to decrease the *TNF* mRNA half-life, which explains some of its therapeutic effects. Using *CRBN* knockdown, it has been shown that the inhibitory effect of IMiDs on TNF-α production was also impaired in the silencing of *CRBN*.^16^ ^,^ ^35^

*CRBN* is composed of 11 exons extending over 30 Kb. Thalidomide binds to CRBN in a region of 104 amino acids (339-442) located in the C-terminal portion, encoded by exons 9, 10 and 11.^13^ This gene is extremely conserved and a few polymorphisms was found in the coding region.^23^ ^,^ ^36^ Studies performed with MM showed that variants in non-coding regions of *CRBN* were associated with response to thalidomide and others IMIDs therapy.^20^ ^,^ ^21^ ^,^ ^37^

The genetic variants evaluated in the present study were identified in a previous study as possible splicing sites.^23^ The association of these SNPs and the dose of thalidomide could indicate that polymorphisms in these regions could interfere in the expression of the *CRBN* gene or in the activity of *CRBN*, being able to modulate the response to treatment with thalidomide. However, in this study, we were unable to identify an association between these variants and the dose variation of thalidomide over time.

We found an association between the genetic variant rs1672770 (p = 0.040) and the manifestation of gastrointestinal adverse effects. There was also an association of gastrointestinal adverse effects (p = 0.015) with haplotype DEL/C/T (rs4183/rs1672770/rs1620675). These adverse gastrointestinal effects consisted of diarrhoea, vomiting, nausea and constipation. The most common symptom was constipation, a commonly reported side effect of thalidomide.^22^ ^,^ ^38^ ^,^ ^39^ The association of adverse effects with the polymorphisms studied may also be related to differences in *CRBN* expression. Mlak et al.^22^ found an association between *CRBN* variants (rs6768972, rs16727) and the risk of polyneuropathy and gastrointestinal disorders in patients with MM treated with thalidomide-based regimens. These variants are located in the *CRBN* promoter region. Thus, they could influence the expression or activity of CRBN.^23^ In the haplotype analysis, only one combination of SNPs showed r2>0.8 (rs4183/rs1620675 with r2 = 0.943), as can be observed in the Supplementary Table. Taking into account this result, our small sample size, and that this is the first study to evaluate CRBN haplotypes, we decided to maintain the logistic regression analysis considering all SNPs. Further studies should be performed in order to elucidate and confirm the haplotypes and associations found here.

In this study, 46 patients were using MDT during the treatment for ENL and only two patients received an alternative MDT regimen. For this reason, they were not divided into a subgroup during the analyses. In addition, the concomitant medications that patients used during treatment for ENL can be diverse. That is why they were not listed in a table. MDT and other drugs were used as covariates in the GEE analysis to avoid bias in the use of these drugs. However, it was not possible to use this correction in the other analyses. The epidemiological data of this study were in agreement with the data found in other studies.^2^ ^,^ ^40^ Most of the individuals with ENL had LL and were male. These frequencies corroborate that men may be more affected by LL and that the bacillary index is a risk factor for the development of the reaction as demonstrated in other studies.^2^ ^,^ ^41^ ^,^ ^42^ In addition, many patients were on MDT during treatment for ENL, confirming that the reaction manifests mainly in the first year of illness, during MDT.^43^ ^,^ ^44^

Some limitations should be considered during the interpretation of our study. It was not possible to obtain samples of skin lesions from these patients. Thus, the DNA samples analysed in this work were obtained from saliva because it presents a methodology that is easier to obtain. However, this does not influence the results presented because it is about the analysis of genetic variants and not the gene expression. Clearly, further studies could focus on the impact of the CRBN expression in the skin lesions before and after the treatment. In addition, the use of clofazimine and in MDT for leprosy, because of its anti-inflammatory effect, and the other drugs may interfere with both the dose reduction of drugs and in the manifestation of adverse effects.^2^ ^,^ ^3^ This is also a retrospective study carried out with clinical data of patients, thalidomide dose, and manifestation of adverse effects obtained through the analysis of medical records of up to six consultations. The lack of standardization in the description of this information or even the absence of a description of the adverse effects cannot be ruled out. Furthermore, as ENL is a chronic condition and its treatment is very long, in this study, the mean treatment time during the collection period was 226 days, but some patients had been in treatment for years and remained on thalidomide treatment after the data collection period. Thus, in some cases, it was not possible to completely monitor the treatment of patients. Peripheral neuropathy, one of the main adverse effects of thalidomide,^3^ ^,^ ^4^ was not evaluated in this study due to its retrospective nature, making it difficult to differentiate neuropathy due to the disease itself or due to the use of thalidomide. Accordingly, the evaluation of the association of variants on the onset of peripheral neuropathy should be performed by a prospective study to reduce potential biases. Another limitation was that this study was carried out with individuals from different regions of the country and the heterogeneous genetic background of the Brazilian population might have been underestimated in the analysis and interpretation of the results. In addition, the limited sample size used in this study can become difficult the identification of minor effects from the genetic variants in different outcomes.

On basis of the results of this study, we identified some genetic variants of *CRBN* were associated with adverse gastrointestinal effects. These results indicate that such variants could impact the protein and influence the outcome of treatment with thalidomide. This also indicates that CRBN may also be necessary for the action of thalidomide in ENL, as already described in MM.^29^ Clearly, more studies evaluating the impact the variants in *CRBN*, as well as associations with ENL treatment, must be performed in order to confirm this hypothesis. ENL is a chronic and difficult-to-control condition. Thalidomide is an effective drug, but has restrictions on its use due to peripheral neuropathy and its teratogenicity.^7^ ^,^ ^8^ Therefore, it is important to identify useful biomarkers in predicting treatment response to limit its use to patients who will benefit most from treatment. To our knowledge, this is the first study to evaluate the association of genetic variants of *CRBN* with thalidomide treatment in ENL patients. There are still many gaps to be filled in our knowledge of the mechanism of action of thalidomide and on how CRBN participates in this process. Thus, this study shows that evaluation of *CRBN* and its expression may help to understand the action of thalidomide in ENL and perhaps, in the future, be a useful biomarker in ENL.
