# Supplementary material for: Evaluation of the influence of genetic variants in Cereblon gene on the response to the treatment of erythema nodosum leprosum with thalidomide
Source: Mem Inst Oswaldo Cruz. 2022 Nov 14;117:e220039. doi: 10.1590/0074-02760220039 (PMC9668341; doi:10.1590/0074-02760220039)
Supplement: Supplementary file 1 [file 1678-8060-mioc-117-e220039-s.pdf]

TABLE

Linkage disequilibrium values (D') of polymorphisms in *cereblon* (*CRBN*) gene

Variant 1	Variant 2	r2
rs4183	rs1672770	0.513
rs4183	rs1620675	0.943
rs1672770	rs1620675	0.567

CC BY
